# Supplementary material for: The Missing Tailed Phages: Prediction of Small Capsid Candidates
Source: Microorganisms. 2020 Dec 8;8(12):1944. doi: 10.3390/microorganisms8121944 (PMC7762592; doi:10.3390/microorganisms8121944)
Supplement: Supplementary file 1 [file microorganisms-08-01944-s001.zip › supplementary_files/Luque_etal_prediction_small_tailed_phages_SM_2020_10_29.pdf]

*microorganisms*

*Supplementary Material*

# The Missing Tailed Phages: Prediction of Small Capsid Candidates

**Antoni Luque[1,2,3,*], Sean Benler[4], Diana Lee [1,2], Colin Brown[1,5], and Simon White[6]**

[1] Viral Information Institute, San Diego State University, San Diego, CA, USA.

[2] Computational Science Research Center, San Diego State University, San Diego, USA.

[3] Department of Mathematics and Statistics, San Diego State University, San Diego, USA.

[4] National Center for Biotechnology Information (NCBI), Bethesda, MD, USA.

[5] Department of Physics, San Diego State University, San Diego, USA.

[6] Department of Molecular and Cell Biology, University of Connecticut, Storrs, CT, USA.

* Correspondence: aluque@sdsu.edu.

Received: date; Accepted: date; Published: date

# Supplementary Figures and Tables

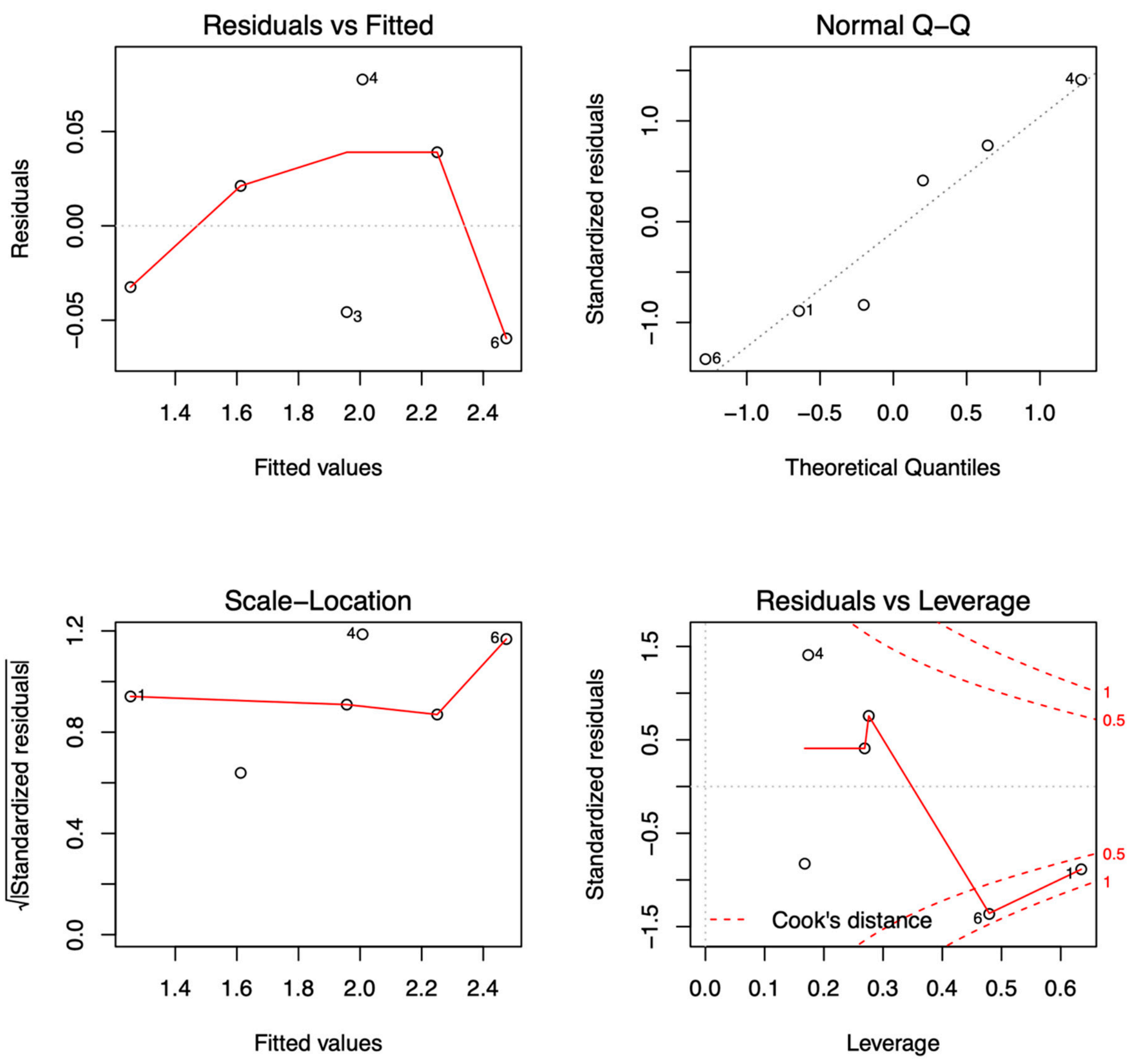


**Figure S1.** Residual diagnostics for the genome length model.

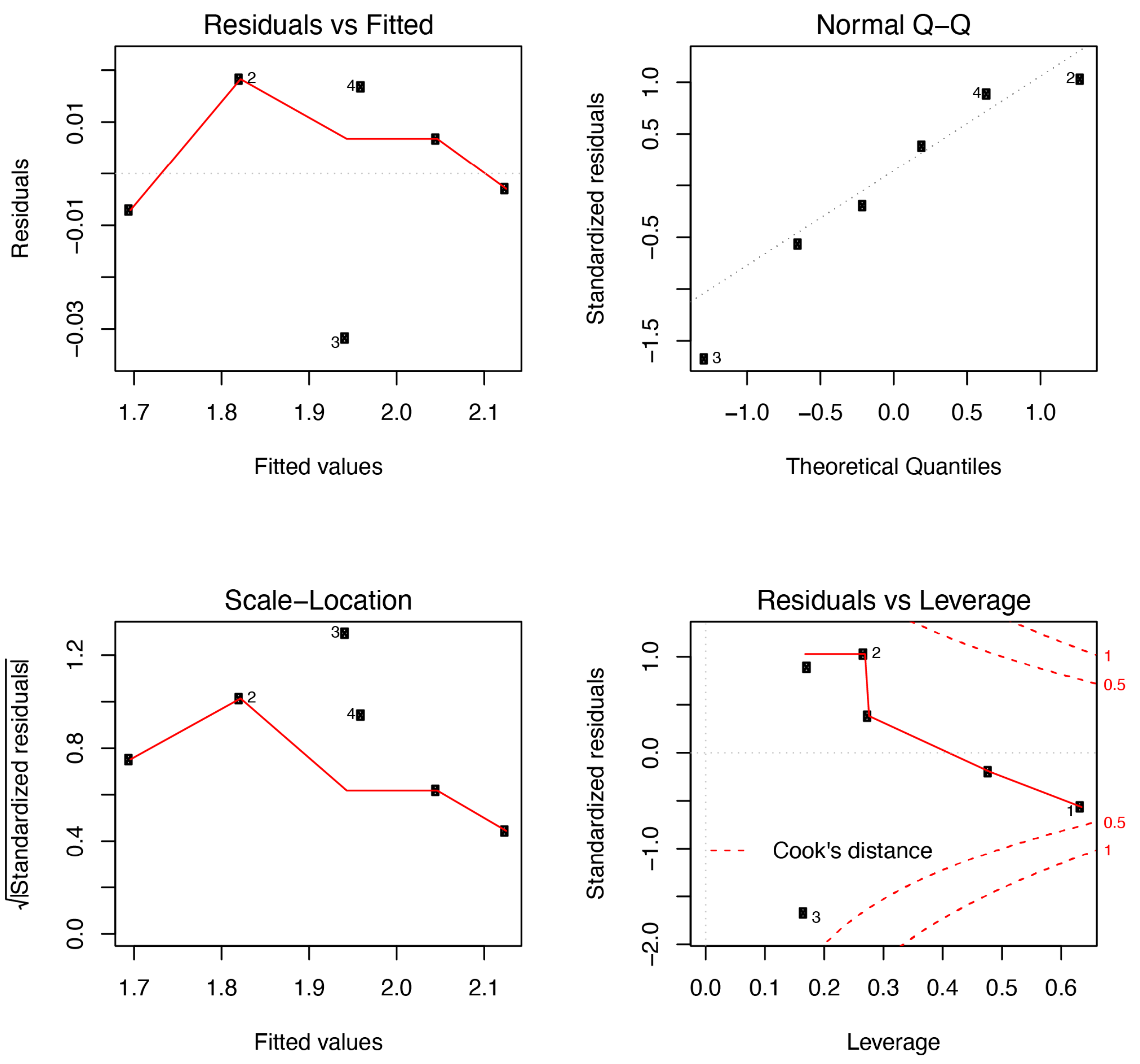


**Figure S2.** Residual diagnostics for the capsid diameter model.

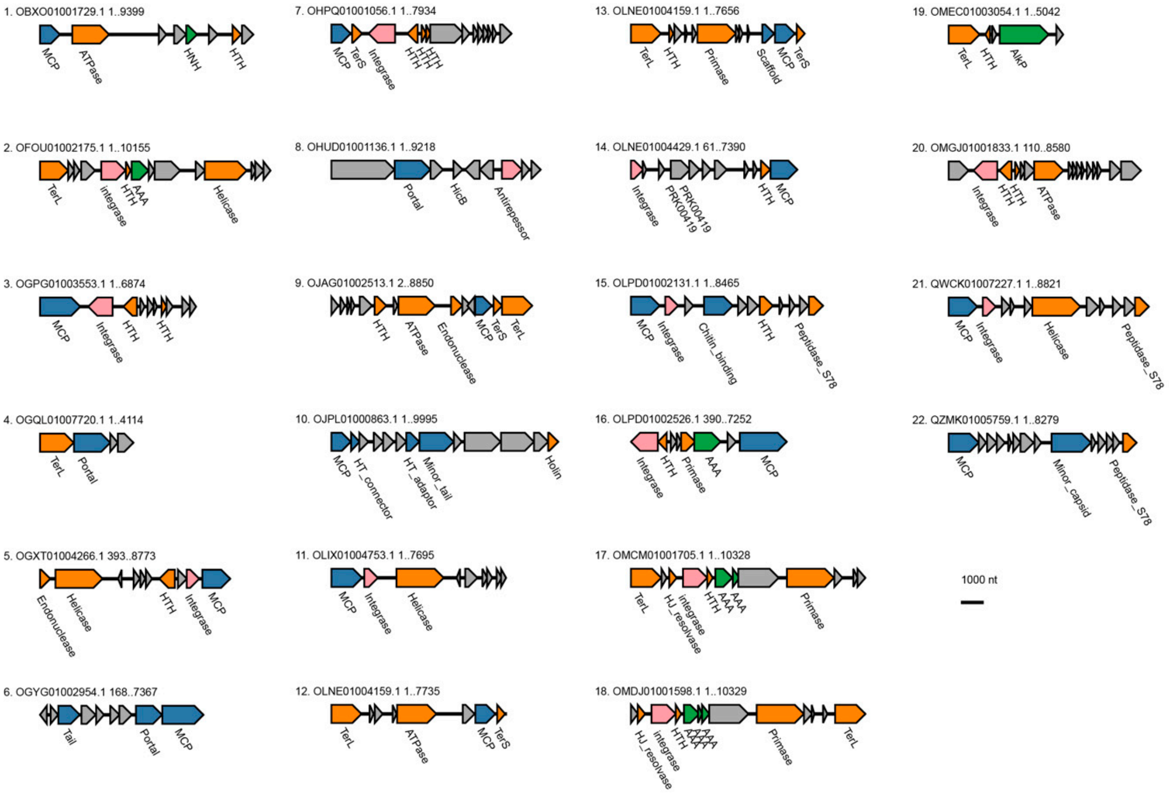


**Figure S3.** Annotated metagenome-assembled tailed phage genomes for predicted T ≤ 3 capsid architectures.

| **Property** | **rho** | **p-value** |
|---|---|---|
| Interior volume | 0.924 | $4.09 \cdot 10^{-6}$ *** |
| Interior surface | 0.923 | $4.08 \cdot 10^{-6}$ *** |
| Exterior surface | 0.989 | $2.84 \cdot 10^{-6}$ *** |
| Genome size | 0.817 | $3.37 \cdot 10^{-6}$ *** |
| Capsids thickness | 0.663 | 0.0010 ** |
| Interior sphericity | –0.566 | 0.0069 ** |
| Exterior sphericity | –0.684 | 0.0006 *** |
| Genome density | –0.162 | 0.4695 |
| MCP interior area§ | 0.484 | 0.0242 * |
| MCP exterior area§ | 0.380 | 0.0819 |

**Table S1.** Correlation analysis with capsid size. Spearman's correlation coefficient (rho) for structural properties as a function of the external capsid diameter. The stars in the p-value are associated to standard cut-offs for statistical significance: (*) $p < 0.05$, (**) $p < 0.01$, and (***) $p < 0.001$.
